# Nordic Walking at Maximal Fat Oxidation Intensity Decreases Circulating Asprosin and Visceral Obesity in Women With Metabolic Disorders

**DOI:** 10.3389/fphys.2021.726783

**Published:** 2021-09-03

**Authors:** Malgorzata Kantorowicz, Jadwiga Szymura, Zbigniew Szygula, Justyna Kusmierczyk, Marcin Maciejczyk, Magdalena Wiecek

**Affiliations:** ^1^Ph.D. Studies, Faculty of Physical Education and Sport, University School of Physical Education in Kraków, Kraków, Poland; ^2^Department of Clinical Rehabilitation, Faculty of Motor Rehabilitation, University School of Physical Education in Kraków, Kraków, Poland; ^3^Department of Sports Medicine and Human Nutrition, Faculty of Physical Education and Sport, Institute of Biomedical Sciences, University School of Physical Education in Kraków, Kraków, Poland; ^4^Department of Physiology and Biochemistry, Faculty of Physical Education and Sport, University School of Physical Education in Kraków, Kraków, Poland

**Keywords:** asprosin, maximum fat oxidation, aerobic training, metabolic syndrome, abdominal obesity, body adiposity index, Nordic walking

## Abstract

**Objective:**

Excess visceral adipose tissue is associated with insulin resistance and other metabolic disorders, including deregulation of adipokine secretion, which may be corrected by aerobic exercise training. Asprosin is a novel adipokine responsible for the regulation of appetite and the release of glucose from the liver, and its levels are pathologically elevated in obesity. The aim of the study was to evaluate the effects of 8-week Nordic walking (NW) training at maximal fat oxidation intensity (FAT_*max*_) on changes in body mass, as well as those in insulin resistance and asprosin levels among young women with visceral obesity and metabolic disorders.

**Materials and Methods:**

The study was completed by 14 women (30.14 ± 3.63 years) representing low levels of physical activity, visceral obesity (waist circumference 105.50 ± 14.87 cm, BMI 33.85 ± 5.48 kg/m^2^) and with metabolic disorders, who for 8 weeks (three times a week, 60 min), participated in NW training at the FAT_*max*_ intensity (61.92 ± 6.71% HR_*max*_, 42.33 ± 8.69% VO_2max_) controlled on the basis of heart rate (114.21 ± 14.10 bpm).

**Results:**

After 4 and 8 weeks of NW training, a significant decrease in the concentration of asprosin, waist and hip circumference (HC), waist-to-height ratio and body adiposity index (BAI) (*p* < 0.05, large effect size) were found.

**Conclusion:**

The 8-week NW training at an FAT_*max*_ intensity decreases the concentration of asprosin in the blood as well as visceral obesity in young women with metabolic disorders.

## Introduction

Excess visceral adipose tissue is associated with the presence of insulin resistance and impaired glucose tolerance, which, if left untreated, lead to the development of Type 2 Diabetes Mellitus (T2DM), while activating pro-thrombotic and pro-inflammatory processes, as well as oxidative stress (Fernández-Sánchez et al., 2011; de Leal and Marfa, 2013; Mahjoub and Roudsari, 2014). According to a report by the International Diabetes Federation (IDF) (2019) 463 million adults (20–79 years) had diabetes worldwide in 2019, of which 4.2 million died from this disease. According to forecasts, the number of patients and diabetes is expected to increase to 578 million by 2030 and to 700 million by 2045. In Europe, in 2045, a 15% increase in the number of diabetics compared to 2019 is expected (International Diabetes Federation (IDF), 2019). Currently, the coexistence of glucose and lipid metabolism disorders, as well as arterial hypertension, which constitute criteria of metabolic syndrome (MetS), are more and more frequently observed, and concern mainly obese and insulin-resistant individuals (Reaven, 1988; McCracken et al., 2018). It is estimated that up to 40% of adults in developed countries have MetS (McCracken et al., 2018). Additional symptoms of MetS include: hyperleptinemia and decreased secretion of adiponectin (Puustinen et al., 2011). The occurrence of MetS increases the risk of T2DM (twofold) (Grundy, 2012), cardiovascular diseases (CVD), including atherosclerosis (fivefold) (Doyle et al., 2012), and furthermore increases overall and CVD-related mortality (Isomaa et al., 2001). In recent studies, it has been indicated that obesity may also contribute to a more severe course of SARS-CoV-2 infection (Popkin et al., 2020).

The pathophysiological mechanisms that link excess white adipose tissue with metabolic disorders are not fully understood. Knowledge about possible complications of MetS is also incomplete and requires further research (Korek and Krauss, 2015; Murawska-Ciałowicz, 2017). Particular interest in adipose tissue was noted with the discovery of numerous compounds having hormonal properties, produced by adipocytes, and are referred to as adipokines (Fernández-Sánchez et al., 2011; Corrêa et al., 2019). Some of them significantly contribute to systemic inflammation, promoting the development of metabolic diseases (de Leal and Marfa, 2013; Francisco et al., 2018).

The most recently described adipokine is asprosin. Asprosin is composed of 140 amino acids and is the C-terminal product of the cleavage of profibrillin encoded by the FBN1 gene (Romere et al., 2016). Romere et al. (2016) showed that asprosin is a hormone secreted into the bloodstream mainly by white adipose tissue adipocytes (Romere et al., 2016). Asprosin regulates glucose release from hepatocytes by activating the protein-G/cAMP/PKA pathway *via* the OLFR734 receptor (Romere et al., 2016; Li et al., 2019). Its highest concentration is recorded in fasting state (Romere et al., 2016). An increase in the concentration of asprosin has impact on the development of insulin resistance and cell apoptosis (Romere et al., 2016; Duerrschmid et al., 2017; Lee et al., 2019). It has been shown that asprosin crosses the blood-brain barrier and is orexigenic (Romere et al., 2016; Duerrschmid et al., 2017), however, in some studies, this has not been confirmed (Jung et al., 2019). On the other hand, the gluconeogenic and appetite-stimulating effects of this cytokine were established in the research by Li et al. (2018).

In overweight individuals, with 1st- and 2nd-degree obesity, and also in those with 3rd-degree obesity, the level of asprosin was 2, 3, and 4 times higher, respectively, compared to the group with normal body mass (Ugur and Aydin, 2019). However, in obese children, the concentration of asprosin in the blood is lower compared to their peers demonstrating body mass within the norm (Long et al., 2019). There was also an increased level of circulating asprosin in fasting state, both in adults and children with insulin resistance (Romere et al., 2016; Wang C.Y. et al., 2019; Wang M. et al., 2019), in people with T2DM (Wang Y. et al., 2018), and in women with polycystic ovary syndrome (Alan et al., 2019). In turn, in neonatal progeroid syndrome (NPS), mutations in the *FBN1* gene occur, leading to asprosin deficiency and lipodystrophy (Romere et al., 2016; Duerrschmid et al., 2017). Thus, the level of asprosin is related to both glucose and lipid metabolism, and lowering it should improve insulin sensitivity.

Worryingly, hypokinesia is the 4th risk factor for death worldwide, contributing to approximately 3.2 million deaths annually (Tian and Meng, 2019), while patients are more likely to adopt a low-calorie diet than to increase physical activity (Chudek, 2009). In order to improve quality of life and prevent chronic diseases such as T2DM or CVD, the American Heart Association recommends 150 min of moderate physical activity or 75 min of vigorous physical exercise per week (Lobelo et al., 2018). For people with high body mass and fat tissue, including those with MetS, workouts are recommended that will additionally relieve the joints of the lower limbs, e.g., aqua-aerobics, hydrospinning, and Nordic walking (NW), which has been increasingly popular in recent years (Liuke et al., 2005; Sanchez-Lastra et al., 2020).

Nordic walking is a form of physical activity requiring the use of specially designed poles, with which the force is pushed away from the ground, resembling the Nordic Skiing style (Hartvigsen et al., 2010; Pellegrini et al., 2015). The main advantage of the NW technique is involvement of muscles not used during standard walking (Shim et al., 2013; Pellegrini et al., 2015), which results in higher energy expenditure. It was found that during NW, oxygen consumption is about 18–25% higher compared to walking without poles at the same speed (Figard-Fabre et al., 2010; Pellegrini et al., 2015). The NW technique makes this possible and the importance of maintaining high-intensity exercise and a low rate of perceived exertion (Figard-Fabre et al., 2010; Pellegrini et al., 2015).

Regular physical activity causes beneficial changes in the human body, even in the absence of weight reduction. However, the influence of physical exercise on the concentration of human asprosin is still little understood (Ceylan and Saygin, 2020). There are no studies regarding the effects of NW on changes in carbohydrate metabolism indices in relation with the influence on asprosin concentration, especially in young overweight/obese people and metabolic disorders.

The aim of the study was to evaluate the effects of 8-week NW training at maximal fat oxidation intensity (FAT_*max*_) on changes in body mass, as well as those in insulin resistance and asprosin levels among young women with visceral obesity and metabolic disorders.

We hypothesize that the concentration of asprosin after 8 weeks of NW training at FAT_*max*_ intensity will decrease, which will further be associated with a simultaneous decrease in insulin resistance among young women with visceral obesity.

## Materials and Methods

### Study Design

The study involved Caucasian women from Central Europe, aged 20–40 years, representing low physical activity (Biernat et al., 2007), visceral obesity and metabolic disorders, who obtained medical qualification for participation in the first phase of the project. During the internal medical examination, medical history was recorded and physical examination was conducted, while MetS was diagnosed in accordance with the 2005 IDF criteria (Alberti et al., 2006), according to which visceral obesity is a prerequisite for the diagnosis of MetS (waist circumference for women in the European population >80 cm), and the presence of two of the four additional factors (for women):

- TG ≥ 1.7 mmol/L or treatment of dyslipidaemia,

- HDL-C < 1.29 mmol/L, or treatment of dyslipidaemia,

- SBP ≥ 130 mmHg or DBP ≥ 85 mmHg or treatment of arterial hypertension,

- GLU ≥ 5.6 mmol/L or diagnosed and treated T2DM.

During cardiological examination, a stress test was performed with simultaneous recording of the electrical activity of the heart (ECG). Based on the results of the cardiological examination, the participants’ ability to perform physical exercise of submaximal and maximal intensity was assessed.

The second phase of the study lasted 16 weeks and included two stages: an 8-week control study without NW control (CON) training and an 8-week NW training period. The order and manner of performing individual test procedures were identical in both stages. NW training was implemented immediately after completion of the CON stage. The results of the final measurements and determinations obtained during the CON stage were, at the same time, the baseline values for the NW stage. The study group was also a control group for itself. In the second phase of the research, the following were evaluated: somatic measurements, graded stress test, biochemical determinations and analysis of diet.

The volunteers were informed in detail about the purpose and plan of the study, and were given information on the method of processing as well as archiving personal data and the obtained results. Participation in the study was voluntary, but required the participants’ written informed consent.

The study was conducted according to the guidelines of the Declaration of Helsinki, and approved by the Bioethical Committee of the Regional Medical Chamber (141/KBL/OIL/2013, 30 December 2013).

### Participants

The projected comprised 78 volunteers. Women with menstrual cycle disorders, those pregnant, in the first year after childbirth, during lactation, using a special diet, including a reduction diet in a period shorter than 6 months before initiation of the study, were excluded. Women with contraindications to exercise at maximal or submaximal intensity in the form of walking or running were also excluded, as well as those using hormonal contraceptives or medications, except for ones MetS-approved by the diagnostic criteria in accordance with the assumptions of the IDF from 2005 (Alberti et al., 2006). The participants declared their eating habits during the study.

Among the participants, 23 subjects meeting the inclusion criteria were enrolled in the study. During the project implementation, nine individuals withdrew from the study (syncope/discomfort with venous blood sampling, lack of training continuity or without justification). Ultimately, the entire study protocol was completed by 14 women, 11 of whom were diagnosed with MetS, and 3 others – women with visceral obesity with 1 coexisting metabolic disorder (Table 1).

**TABLE 1:** Number of persons meeting individual criteria of metabolic syndrome (MetS) in accordance with 2005 IDF assumptions (Alberti et al., 2006).

Variable	WC (cm)	HDL-C (mmol/l)	TG (mmol/l)	GLU (mmol/l)	SBP (mmHg)	DBP (mmHg)
MetS criterion acc. to IDF	>80	<1.29	≥1.7	≥5.6	≥130	≥85
Number of persons (*n*)	14	8	3	4	10	8
Percentage of persons (%)	100	57	21	29	71	57

*WC, waist circumference; HDL-C, high-density lipoproteins; TG, triglycerides; GLU, glucose; SBP, systolic blood pressure; DBP, diastolic blood pressure.*

The mean age of the examined women (*n* = 14) was 30.14 ± 3.63 years. The subjects’ body mass (BM) and body mass index (BMI) were, on average, 92.10 ± 17.99 kg (67.8–134.0 kg) and 33.85 ± 5.48 kg/m^2^ (26.44–45.83 kg/m^2^). Blood morphology as well as systolic (SBP) and diastolic blood pressure (DBP) of the subjects are presented in Table 2 while metabolic characteristics of the subjects are presented in Table 3.

**TABLE 2:** Morphology, systolic (SBP), and diastolic blood pressure (DBP) of the subjects.

Variable	Mean ± SD	Me (Q1, Q3)	Min-Max	Norms
Erythrocytes (10^6^/μL)	4.65 ± 0.33	4.56 (4.41, 4.88)	4.20–5.31	3.5–5.0
Haemoglobin (g/dL)	13.60 ± 0.71	13.70 (13.20, 14.00)	12.10–14.80	11.0–15.0
Haematocrit (%)	40.06 ± 2.01	40.25 (38.40, 41.50)	36.20–43.40	37.0–47.0
Leukocytes (10^3^/μL)	7.30 ± 1.96	6.68 (6.21, 7.46)	5.26–12.79	4.0–10.0
Neutrophils (%)	57.02 ± 8.66	56.65 (50.70, 63.50)	39.20–71.00	58.0–66.0
Eosinophils (%)	2.68 ± 2.05	2.25 (1.00, 3.10)	1.00–7.00	1.0–3.0
Basophils (%)	0.64 ± 0.43	0.90 (0.20, 1.00)	0.00–1.00	0.0–1.0
Lymphocytes (%)	31.34 ± 7.78	29.00 (25.10, 36.40)	21.60–48.00	20.0–40.0
Monocytes (%)	8.54 ± 2.44	8.00 (7.00, 9.00)	6.00–14.00	4.0–8.0
Thrombocytes (10^3^/μL)	285.36 ± 46.66	269.0 (258.0, 333.0)	224.0–373.0	125.0–340.0
SBP (mmHg)	136.79 ± 12.80	140.0 (130.0, 140.0)	110.0–160.0	120.0–129.0
DBP (mmHg)	86.79 ± 7.23	87.5 (80.0, 90.0)	80.0–90.0	80.0–84.0

*SD, standard deviation; Me, median; Q1, lower quartile; Q3, upper quartile; SBP, systolic blood pressure; DBP, diastolic blood pressure.*

**TABLE 3:** Metabolic features of the subjects.

Variable	Mean ± SD	Me (Q1, Q3)	Min–Max	Norms
TC (mmol/L)	4.68 ± 1.04	4.57 (4.10, 5.50)	2.75–6.67	<5.2
LDL-C (mmol/L)	2.72 ± 0.96	2.40 (2.40, 3.45)	1.13–4.92	<3.0
HDL-C (mmol/L)	1.35 ± 0.30	1.25 (1.15, 1.58)	0.93–1.90	>1.2
TG (mmol/L)	1.19 ± 0.61	1.16 (0.75, 1.37)	0.29–2.53	0.2–1.7
GLU (mmol/L)	5.63 ± 2.34	5.11 (4.48, 5.60)	4.11–13.52	3.4–5.5
HbA1c (%)	5.35 ± 1.31	5.14 (4.94, 5.43)	3.56–9.60	4.3–5.9
INS (μU/mL)	15.20 ± 6.86	16.35 (7.30, 17.20)	6.10–29.20	2–15
HOMA-IR	3.82 ± 2.26	3.90 (1.62, 4.35)	1.11–10.03	<2.5
Quicki	0.32 ± 0.03	0.31 (0.31, 0.36)	0.28–0.38	>0.34
TyG	8.40 ± 0.68	8.46 (7.89, 8.72)	7.27–10.01	None
Asprosin (ng/mL)	14.38 ± 15.99	8.59 (4.18, 12.42)	2.37–47.26	None
LEPT (ng/mL)	26.53 ± 8.47	29.46 (22.75, 31.50)	9.22–36.60	7–13
ADIPO (μg/mL)	12.62 ± 5.38	11.16 (8.36, 17.83)	6.37–25.13	5–30
LEPT/ADIPO	2.38 ± 1.15	2.50 (1.45, 2.82)	0.79–5.12	None

*SD, standard deviation; Me, median; Q1, lower quartile; Q3, upper quartile; TC, total cholesterol; LDL-C, low-density lipoproteins; HDL-C, high-density lipoproteins; TG, triglycerides; GLU, glucose; HbA1c, glycated haemoglobin; INS, insulin; HOMA-IR, homeostatic model assessment; Quicki, quantitative insulin sensitivity check index; TyG, triglyceride glucose (lnTG × GLU/2) index; LEPT, leptin; ADIPO, adiponectin.*

### Graded Stress Test

The test was performed immediately prior to the start of the NW stage on a mechanical treadmill (h/p/Cosmos Saturn COS 10198, Nussdorf–Traunstein, Germany). The test began with a 3-min walk at a speed of 3.5 km/h with a treadmill inclination angle of 1°. Then, every 3 min, the belt speed was increased by 0.8 km/h until the respiratory exchange ratio (RER) value was 1.0, and following, every 2 min until exhaustion. The highest heart rate and oxygen uptake noted in this test was considered as maximal heart rate (HR_*max*_) and maximal oxygen consumption (VO_2max_).

The mass of oxidised fat in consecutive minutes of the exercise was estimated for each participant using the formula (Frayn, 1983; Tan et al., 2012), (Eq. 1):

(1) $$F\,A\,T\,\left(g\right)=1.67\,\times {\text{VO}}_{2}\,\,\left(\frac{\text{L}}{\text{min}}\right)-\,1.67\,\times {\text{VCO}}_{2}\,\,\left(\frac{\text{L}}{\text{min}}\right)$$

The highest value was designated as FAT_*max*_. Then, training HR (HR_*NW*_) was determined for the work intensity at which FAT_*max*_ was achieved.

### Nordic Walking Training

Immediately before beginning NW training, the walking technique was introduced, based on the guidelines of the Polish Federation of Nordic Walking (PFNW) and the International Nordic Walking Federation (INWA; Pellegrini et al., 2018). The training of the NW technique (classic technique, so-called health level) was carried out by a qualified instructor.

Nordic walking trainings were conducted three times a week for 8 weeks, under the supervision of an instructor, in flat, green urban areas, at the same time of the day and year. In order to provide better instructor care, the study group was divided into groups of 2–3 participants depending on exercise capacity.

Each training session lasted 60 min (5-min warm-up, 50 min of NW, and 5-min breathing exercises). The mean HR_*NW*_ was 114.21 ± 14.10 bpm, which totalled 61.92 ± 6.71% HR_*max*_ and 42.33 ± 8.69% VO_2max_. Average FAT_*max*_ in the study group was 0.38 ± 0.11 g/min.

Participants controlled the designated HR_*NW*_ with a heart rate monitor (Polar Elektro S610i, Kempele, Finland). A change of the set HR_*NW*_ value by more than ± 10 beats were signalled acoustically, providing information that walking speed needed to be corrected.

### Somatic Measurements

Waist (WC) and hip circumference (HC) (Seca 201, Hamburg, Germany), as well as body mass (Jawon IOI-353 Body Composition Analyzer, Gyeongsan, South Korea) were measured five times: before beginning the study, after 4 and 8 weeks in CON and after 4 and 8 weeks in the NW group. WC and HC were measured in a standing position to the nearest 1 mm, placing an anthropometric tape perpendicular to the vertical axis of the body. WC was measured at the end of calm exhalation, midway between the inferior margin of the ribs and the superior border of the iliac crest in midaxillary line. HC was measured above the buttocks at the widest point around the greater trochanter, making sure not to be lower than the pubic symphysis. All measurements of circumferences were taken three times by the same person with extensive experience, always using the same anthropometric tape. In the analysis, the average of the two closest results was taken into account (Alberti et al., 2006; Ahmad et al., 2016). Before starting the CON, body height (BH) was measured (Seca 231 stadiometer, Hamburg, Germany).

Body mass index, waist-to-hip ratio (WHR), waist-to-height ratio (WHtR), and body adiposity index (BAI) were calculated for each participant according to the formulas (Eqs 2–5):

(2) $$B\,M\,I=B\,M\,\left(k\,g\right)/\text{BH}\,{\left(\text{m}\right)}^{2}$$

(3) W⁢H⁢R=WC⁢(cm)/HC⁢(cm)

(4) W⁢H⁢t⁢R=WC⁢(cm)/BH⁢(cm)

(5) $$B\,A\,I=H\,\text{C}\,\left(\text{cm}\right)/{\left(\text{BH}\,\left(\text{cm}\right)\right)}^{1.5}-18$$

### Biochemical Determinations

Venous blood was collected in fasting state five times: before beginning the study, after 4 and 8 weeks (CON) and after 4 and 8 weeks (NW). The blood was collected from the inner elbow vein, taking the principles of asepsis into account (BD Vacutainer vacuum system, Becton Dickinson, Franklin Lakes, NY, United States). Blood collected for glucose concentration (K2EDTA and glycolysis inhibitors: sodium fluoride and potassium oxalate) and asprosin (K2EDTA and protease inhibitor: aprotinin 0.6 TIU/1 mL of blood) was centrifuged (RCF 1,000 × *g*) immediately after collection for 15 min at 4°C (MPW-351R, MPW Med. Instruments, Warsaw, Poland). For the determination of insulin, clotting activator tubes were used, stored for 20 min at 20–22°C until a clot was formed, and then, centrifuged under the above conditions. The obtained plasma and serum were stored at −70°C until analysis (POL-EKO-APARATURA ZLN-UT 300 PREM low-temperature freezer, Wodzisław Śląski, Poland).

#### Asprosin

The concentration of asprosin in the blood plasma was determined *via* the enzyme immunoassay (ELISA) method with the Nori^®^Human Asprosin ELISA Kit GR 111426 (Genorise, Glen Mills, PA, United States). The detection range was 1.5–100 ng/mL, intra-assay CV <6% and inter-assay CV <9%. The Spark^®^ multimode microplate reader (Tecan, Grödig, Austria) was used to measure absorbance.

#### Carbohydrate Metabolism Indices

The concentration of glucose in the blood plasma was performed *via* the enzymatic method using the Cobas c701/702 biochemical analyser (Roche Diagnostics International Ltd., Mannheim, Germany). Serum insulin concentration was determined by electrochemiluminescence (ECLIA) using the Cobas e801 apparatus (Roche Diagnostics International Ltd., Mannheim, Germany). The determinations were performed according to manufacturer guidelines with the use of reagents dedicated to the GLUC3 and Elecsys Insulin analysers, respectively. The measuring range for the glucose (GLUC3) test was 2–750 mg/dL, while for insulin (Elecsys Insulin), this totalled 0.4–1000 mU/mL. The following indices were calculated accordingly (Eqs 6, 7):

(6) $$H\,O\,M\,A-I\,R=\frac{I\,N\,S\,\left(\mu \,\text{I}\,\text{U}/\text{mL}\right)\,\times \text{GLU}\,\left(\text{mmol}/\text{L}\right)}{22.5}$$

(7) $$Q\,U\,I\,C\,K\,I=\frac{1}{\log\text{INS}\,\left(\mu \,\text{I}\,\text{U}/\text{mL}\right)+\log\text{GLU}\,\left(\text{mmol}/\text{L}\right)}$$

### Evaluation of Physical Activity and Nutritional Behaviour

Before beginning the project, the participants’ levels of physical activity were assessed using the International Physical Activity Questionnaire (IPAQ) – Polish version (Biernat et al., 2007). Dietary habits were monitored five times using 3-day food diaries. The subjects entered information based on the mass of consumed products, dishes and drinks on their own, providing values in home- or commercial-units. The serving size was assessed subjectively by the surveyed women on the basis of the Album of Product and Food Photography (Szponar et al., 2000). The diet 6.0 computer program (Food and Nutrition Institute, Warsaw, Poland) was used to evaluate diet. Nutrition assessment was carried out by a qualified dietician at weeks 1, 4, and 8 (CON) and 4 and 8 (NW).

### Statistical Analysis

The distribution of variables was checked with the Shapiro–Wilk test. Data are presented as arithmetic mean ± SD or as median (Me), and lower and upper quartiles (Q1 and Q3). The impact of NW on the level of the analysed variables was assessed using the non-parametric Friedman and Wilcoxon tests. Effect sizes for Wilcoxon analyses were calculated based on η^2^ = z^2^/*n* (*n* = 14, number of persons in group) and interpreted as no effect if η^2^ < 0.01, small effect if 0.01 ≤ η^2^ < 0.09, moderate effect if 0.09 ≤ η^2^ < 0.25, and large effect if η^2^ ≥ 0.25 (Fritz and Morris, 2012). The results of these analyses are presented in tabular form as medians (Q1–Q3). Correlations between the variables were determined (Spearman’s test). The differences in the results and correlations were considered statistically significant for *p* < 0.05. The STATISTICA 13.1 PL for Windows package (StatSoft, Inc., Tulsa, OK, United States) was implemented for statistical calculations.

## Results

### Asprosin

**FIGURE 1:**
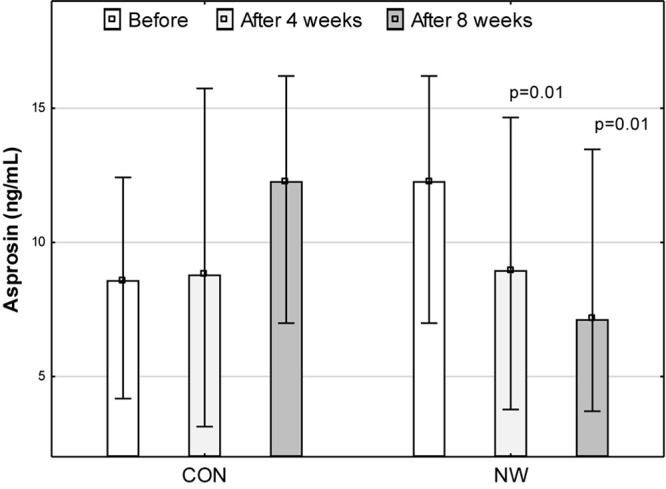
Asprosin concentration in blood plasma during the control (CON) tests and the Nordic walking stage (NW). Data are presented as medians (Me) and quartiles (Q1, Q3); *p* < 0.05 – statistically significant difference with regard to baseline value at a given stage.

Prior to research, the median concentration of asprosin was 8.59 (4.18, 12.42) ng/mL. During the CON stage, the concentration of asprosin did not change significantly. After 4 weeks of CON, the concentration of asprosin was comparable (*p* = 0.40, η^2^ = 0.05) to baseline concentration and totalled 8.80 (3.13, 15.74) ng/mL. After 8 weeks of CON, despite a lack of statistical significance (*p* = 0.16), moderate effect size (η^2^ = 0.14) demonstrated an increase in the level of this hormone to the level of 12.29 (6.99, 16.21) ng/mL. After 4 weeks of NW, the concentration of asprosin decreased significantly (large effect size) by 27.0% (*p* = 0.01, η^2^ = 0.56), while after 8 weeks, the level of this hormone was lower by 41.9% (*p* = 0.01, η^2^ = 0.51) relative to the baseline value (large effect size). The median concentration of asprosin was 8.97 (3.77, 14.66) ng/mL and 7.14 (3.70, 13.47) ng/mL after 4 and 8 weeks of NW, respectively (Figure 1).

### Glucose Metabolism Indices

During CON and NW, no statistically significant changes (*p* ≥ 0.05) were noted for GLU, INS levels, or in HOMA-IR and Quicki values (Table 4).

**TABLE 4:** Blood glucose and insulin levels as well as level of insulin resistance indices during the control (CON) and Nordic walking (NW) stages.

Variable	Stage	Before stage	After 4 weeks	After 8 weeks	Effects of intervention		

		1	4	8	Friedman’s test	d4-1	d8-1
		Me (Q1, Q3)	Me (Q1, Q3)	Me (Q1, Q3)	*p*	*p* (η ^2^)	*p* (η ^2^)
**GLU (mmol/L)**	CON	5.11 (4.48, 5.60)	5.04 (4.76, 5.22)	5.02 (4.78, 5.54)	0.61	0.16 (0.14)	0.92 (<0.01)
	NW	5.02 (4.78, 5.54)	5.07 (4.70, 5.46)	5.12 (4.74, 5.35)	0.61	0.55 (0.03)	0.85 (<0.01)
**INS (μU/mL)**	CON	16.35 (7.30, 17.20)	13.20 (10.10, 15.80)	12.95 (10.50, 17.70)	0.93	0.55 (0.03)	0.78 (<0.01)
	NW	12.95 (10.50, 17.70)	13.65 (11.10, 15.80)	14.55 (9.40, 17.00)	0.81	0.97 (<0.01)	0.90 (<0.01)
**HOMA-IR**	CON	3.90 (1.62, 4.35)	2.80 (2.14, 3.98)	3.07 (2.40, 3.83)	0.93	0.60 (0.03)	0.59 (0.02)
	NW	3.07 (2.40, 3.83)	3.28 (2.54, 3.77)	3.44 (2.22, 4.00)	0.81	0.92 (<0.01)	0.97 (<0.01)
**Quicki**	CON	0.31 (0.31, 0.36)	0.33 (0.31, 0.34)	0.32 (0.31, 0.33)	0.98	0.78 (<0.01)	0.97 (<0.01)
	NW	0.32 (0.31, 0.33)	0.32 (0.31, 0.33)	0.32 (0.31, 0.34)	0.93	0.94 (<0.01)	0.97 (<0.01)

*Me, median; Q1, lower and (Q3) upper quartile; d4-1 and d8-1, difference in results obtained after 4 and 8 weeks, respectively; and before beginning given stage, *p* < 0.05, statistically significant difference; *p* ≥ 0.05, statistically insignificant difference; η^2^, effect size: none η^2^ < 0.01, small 0.01 ≤ η^2^ < 0.09, moderate 0.09 ≤ η^2^ < 0.25, large η^2^ ≥ 0.25, GLU, glucose, INS, insulin, HOMA-IR = INS (μIU/mL) × GLU (mmol/L)/22.5, QUICKI = 1/[log INS (μIU/mL) + log GLU (mg/dL)].*

### Anthropometric Indices

During the CON period, no statistically significant differences were found in BM, BMI, WC, HC, or in the values of anthropometric indexes: WHR, WHtR, and BAI (*p* ≥ 0.05) (Table 5).

**TABLE 5:** Somatic characteristics of participants during the control (CON) and Nordic walking (NW) stages.

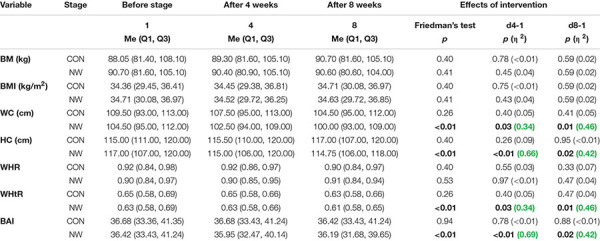

*Me, median; Q1, lower and (Q3) upper quartile; d4-1 and d8-1, difference results obtained after 4 and 8 weeks; and beginning given stage, *p* < 0.05, statistically significant difference; *p* ≥ 0.05, statistically insignificant difference; η^2^, effect size: none η^2^ < 0.01, small = 0.01 ≤ η^2^ < 0.09, moderate 0.09 ≤ η^2^ < 0.25, large η^2^ ≥ 0.25 (green), BM, body mass; BMI, body mass index; WC, waist circumference; HC, hip circumference; WHR, waist-to-hip ratio; WHtR, waist-to-height ratio; BAI, body adiposity index. Bold values are statistically significant differences.*

**FIGURE 2:**
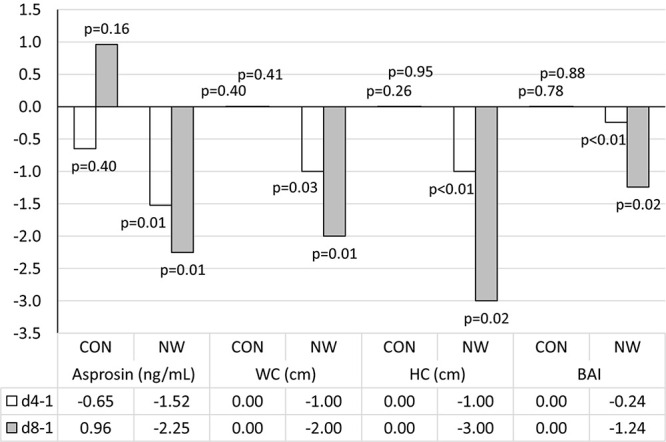
Changes in analysed variables after 4 (d4-1) and 8 weeks (d8-1) of control (CON) tests and the Nordic walking (NW) compared to values at the beginning of the stage. Data are presented as medians (Me); *p* < 0.05 – statistically significant difference with regard to baseline value at a given stage; WC, waist circumference; HC, hip circumference; BAI, body adiposity index.

After 4 weeks of NW, a statistically significant decrease in WC (*p* = 0.03, η^2^ = 0.34) and HC (*p* < 0.01, η^2^ = 0.66) was noted, as well as a reduction in the values of WHtR (*p* = 0.03, η^2^ = 0.34) and BAI (*p* < 0.01, η^2^ = 0.69), compared to the baseline values. In each of these cases, a large effect size was found (Table 5 and Figure 2).

After 8 weeks of NW, it was demonstrated that both WC (*p* = 0.01, η^2^ = 0.46) and HC (*p* = 0.02, η^2^ = 0.42) were significantly smaller compared to baseline (large effect size). After 8 weeks of NW, significantly lower WHtR (*p* = 0.01, η^2^ = 0.46), BAI values (*p* = 0.02, η^2^ = 0.42) were noted compared to baseline, indicating a large clinical effect (Table 5 and Figure 2).

During NW, however, no statistically significant changes in BM, BMI, or WHR were noted, the level of which throughout the study period was similar to the baseline (Table 5).

### Mutual Dependencies Between the Analysed Variables

No significant correlations were found between the level of asprosin and the analysed variables (*p* ≥ 0.05). There was a significant, negative correlation between the concentration of adiponectin and the LEPT/ADIPO index (*r* = −0.60, *p* < 0.05), INS (*r* = −0.57, *p* < 0.05) and HOMA-IR (*r* = −0.55, *p* < 0.05), as well as a positive correlation with HDL-C concentration (*r* = 0.65, *p* < 0.05). Leptin concentration indicated a positive correlation with BM (*r* = 0.54, *p* < 0.05) and BAI (*r* = 0.58, *p* < 0.05), while the LEPT/ADIPO ratio showed a positive correlation with leptin concentration (*r* = 0.54, *p* < 0.05), INS (*r* = 0.78, *p* < 0.05), HOMA-IR (*r* = 0.65, *p* < 0.05) and BMI (*r* = 0.55, *p <* 0.05). BAI demonstrated a positive correlation with BM (*r* = 0.63), BMI (*r* = 0.83) and GLU (*r* = 0.54), as well as a negative correlation with HDL-C (*r* = −0.85).

## Discussion

### Influence of Training on Asprosin Concentration

The results of our research were the first to show changes in the concentration of asprosin as a result of 8-week NW training carried out at the intensity of FAT_*max*_. After a series of 24 NW training sessions, we found a significant decrease in the concentration of asprosin among the group of young women with visceral obesity and metabolic disorders, which began to be noticed already in the middle of the training period, and the size of the clinical effect of these changes was large. The intensity of exercise at the FAT_*max*_ level recommended for overweight and obese individuals is within the range of 40–50% VO_2max_, and is considered adequate to maximise the effects of exercise without risking muscle or joint damage (Chávez-Guevara et al., 2020). The applied training in the form of NW with the intensity at which the maximal oxidation of fatty acids occurred, did not affect the level of glucose, insulin or insulin resistance indices (HOMA-IR and Quicki) in the studied group.

We did not find any correlations between the level of asprosin and the level of carbohydrate metabolism indices. A positive correlation between the level of this hormone and the level of glucose in the blood was demonstrated by Wiecek et al. (2019) among a group of obese postmenopausal women, regardless of whether they had MetS or not. In another research trial, a positive correlation was noted between the level of asprosin and HOMA-IR, fasting glycaemia, waist circumference and the level of TG in adult patients with T2DM and impaired glucose tolerance (Wang Y. et al., 2018). In a group of obese children with insulin resistance, there was a positive correlation between the level of asprosin and WHR, HOMA-IR and the ratio of leptin to adiponectin (Wang M. et al., 2019). The results of our study did not allow to indicate such relationships in young women with visceral obesity and metabolic disorders.

There was a decreased level of asprosin in men as a result of single aerobic exercise, and the reduction of this index among obese and overweight men was greater than in the case of men with normal body composition (Ceylan et al., 2020). Schumann et al. (2017), under the influence of single aerobic exercise until exhaustion, did not observe any changes in the level of asprosin among obese women or men and women with normal body mass. On the other hand, Wiecek et al. (2018) noted an increase regarding the level of asprosin in the blood plasma of young women undergoing anaerobic exercise, without obtaining a positive correlation between the level of asprosin and the level of fasting glucose in young people. These studies allow to indicate a diversified response in terms of exercise changes for asprosin concentration depending on gender, body fat level and exercise energy (Schumann et al., 2017; Wiecek et al., 2018; Ceylan et al., 2020). To date, in literature on the subject, there are no studies on changes in the level of asprosin as a result of aerobic training in a group of young women with metabolic disorders. In the presented study, this is the first to analyse such results. The obtained results, despite the lack of changes in body mass, indicate a beneficial effect of low-intensity training on the change in the level of asprosin among women with abdominal obesity.

### Influence of Training on Anthropometric Indices and Metabolic Effects

Increasingly, in clinical practice, apart from direct assessment of body mass, BMI and adipose tissue content, anthropometric indices such as WHR, WHtR, and BAI are used, allowing indirect assessment of disproportions in the content of visceral adipose tissue, as well as the risk of MetS, CVD, or pre-diabetes (Amato et al., 2010; Dong et al., 2017; Koszowska and Broñczyk-Puzoñ, 2018). In our study, after 4 weeks of NW, positive somatic changes were noted in the examined women, which showed a reduction in visceral obesity. There was a decrease in waist and HC, as well as a reduction in anthropometric indices reflecting the degree of body fat, linking changes in the above-mentioned circumferences with BH, i.e., WHtR and BAI. The clinical significance of these changes was high. Extending the training stage to 8 weeks resulted in intensification of these effects. However, during the entire training phase, we did not observe any significant changes in body mass, BMI or WHR.

A WHtR value ≥ 0.5 indicates an increased risk of metabolic diseases (Broñczyk-Puzoñ et al., 2018). All individual WHtR results in our study were higher than the norm, and although they were not fully normalised under the influence of NW, the value of this index did not decrease in only one person.

The assessment of body fat *via* BAI is correlated with the results obtained during the body composition test with the use of Dual Energy X-ray Absoptiometry (DEXA) (Vinknes et al., 2013). BAI has been proposed as a reliable research tool in the American adult population (Bergman et al., 2011). The BAI value in our study was similar to that found in other obese Caucasian women (Jabłonowska-Lietz et al., 2017; Blus et al., 2019), but higher than that for obese Asian women with metabolic disorders (Dong et al., 2017). It has been previously shown that the BAI was the best correlated with leptin (Melmer et al., 2013). In our research, we found a high correlation of BAI with BMI and BM, as well as with the concentration of glucose and leptin, and a negative correlation of this index with HDL-C concentration.

Similar to the results obtained in our study, 8-week endurance and strength training resulted in a significant reduction of BAI and WHtR in older, overweight women (Faramarzi et al., 2018). While in the 8-week study by Tibana et al. (2013), no changes in the level of BAI or WHtR were found in the group of overweight women, but as a result of resistance training.

Contrary to our research, the results obtained by other teams indicate that various forms of physical activity, including NW, carried out at an intensity corresponding to FAT_*max*_, may reduce body mass and have a positive effect on its composition in overweight and obese individuals (Dumortier et al., 2003; Tan et al., 2012; Botero et al., 2013; Besnier et al., 2015; Cebula et al., 2020).

Besnier et al. (2015), in a 5-month project involving young women (20–40 years) with increased body mass, training on a cycloergometer with a FAT_*max*_ intensity of about 45% of VO_2max_ (four times a week, 55 min), found significant decrease in body mass and fat tissue as well as BMI. Furthermore, applying a training period of 8 weeks, as in our study, Tan et al. (2012) reported a decrease in body mass, BMI, mass and percentage of adipose tissue as well as WHR in young women (20–23 years old). Jogging training sessions were performed five times a week for 60 min, their intensity was about 54% VO_2max_, and the maximum fat burning was 0.43 g/min (Tan et al., 2012). A reduction in body mass, BMI and waist circumference was also reported in the research by Botero et al. (2013) conducted among women aged 27–46 with excess body mass after 12 weeks of training on a cycloergometer (three times a week, 60 min). Also, the implementation of NW training at the intensity of FAT_*max*_ caused a reduction in body mass and BMI, but in the group of older women (over 55 years of age) who were overweight, and these changes were recorded in a shorter time than in our study, because they were the result of 6-week training (three times a week, 90 min) (Cebula et al., 2020). The study by Dumortier et al. (2003) included women and men with MetS above the age of 50. They performed 8 weeks of exercises on a cycloergometer at an intensity corresponding to FAT_*max*_ (three times a week, 40 min). There was a significant reduction in body mass, fat percentage and BMI. Similar to the results of our own research, waist and HC in people with MetS decreased significantly, while the WHR index did not change significantly (Dumortier et al., 2003).

The impact of training carried out at the intensity of FAT_*max*_ on the indices of carbohydrate metabolism in the group of people with excess body mass is ambiguous. A significant reduction in the HOMA-IR index was achieved as a result of cycling training (Dumortier et al., 2003; Besnier et al., 2015), which is not confirmed in the results of our research with regard to NW training. On the other hand, similarly to our study, Botero et al. (2013) did not obtain significant differences in glucose levels, while Dumortier et al. (2003) achieved no changes in glucose or insulin levels as a result of training. Besnier et al. (2015) achieved a significant reduction in insulin levels as a consequence of training on a cycloergometer.

Contrary to our research, other teams (Figard-Fabre et al., 2011; Sentinelli et al., 2015; Pippi et al., 2020) indicated significant reductions in body mass and BMI among women and men with increased body mass due to NW training, but at intensity higher than FAT_*max*_. The research results, however, are not unequivocal. In the research carried out by many other teams, as in our trial, no significant effect of NW training on changes in body mass or BMI were found, despite the greater training intensity, regardless of the training period duration (Latosik et al., 2014; Trabka et al., 2014; Wiklund et al., 2014).

In a 6-week study by Wiklund et al. (2014), no changes in body mass or BMI were found as a result of NW training performed with gradually increasing intensity from 60 to 75% of HR_*max*_ in a group of overweight and obese women aged 20–50 years (3–4 times a week, 30–60 min). Contrary to our study, researchers noted a significant reduction in glucose and insulin levels (Wiklund et al., 2014). Also, 8-week NW training at increasing intensity within the range of 40–70% HR_*max*_, carried out in the group of overweight women at a postmenopausal age did not significantly reduce body mass, BMI or WHR, despite the implementation of proper nutrition throughout the study period (Latosik et al., 2014). Trabka et al. (2014) did not find any changes in body mass or BMI, nor in waist and HC, among obese postmenopausal women using 10-week NW training, which was an aerobic part of the aerobic-strength training programme. NW sessions were performed three times a week for 40 min, and their intensity increased every 2 weeks by 10%, within the range of 50–80% heart rate reserve (Trabka et al., 2014).

Analysing the obtained results, it should be emphasised that the results of the meta-analysis clearly indicate that a reduction in body circumference, even if it is not accompanied by changes in body mass and BMI, reduces the risk of CVD, furthers obesity development and related metabolic changes (Freedland, 2004).

The results of our research allow to indicate that, already in the middle of the training period, i.e., after 4 weeks of NW training conducted at an intensity corresponding to FAT_*max*_, there was significant reduction in the level of asprosin, i.e., the hormone responsible, among others, for the release of glucose from hepatocytes, and prolonging the training period exacerbates these changes. However, this time was insufficient to achieve results in terms of lowering glucose and insulin levels or reducing insulin resistance. It is likely that extending the training period could cause such changes. In addition, after the applied NW training, a significant reduction in BAI, as well as waist and HC and WHtR index, was noted, but without changes in body mass, BMI and WHR. The results of our research demonstrate that despite the lack of changes in body mass, low-intensity physical training reduces visceral obesity, and thus, limits the further development of metabolic disorders.

Research on the impact of aerobic training concerning changes in asprosin levels among individuals with metabolic disorders should be continued, extending the training period and including men in the research.

## Conclusion

The 8-week training programme at maximal fat oxidation intensity decreases the concentration of asprosin in the blood as well as visceral obesity in young women with metabolic disorders.

## Data Availability Statement

The raw data supporting the conclusion of this article will be made available by the authors, without undue reservation.

## Ethics Statement

The studies involving human participants were reviewed and approved by the Bioethical Committee of the Regional Medical Chamber (141/KBL/OIL/2013, 30 December 2013) and was conducted according to the guidelines of the Declaration of Helsinki. The patients/participants provided their written informed consent to participate in this study.

## Author Contributions

MW, MK, MM, and ZS planned and designed the study. MK recruited the participants and supervised the exercise intervention and dietary intake data. MK, MW, and JS collected the data. JS and MM processed the exercise test. MK, MW, and ZS processed anthropometric, body composition, and metabolic data. MK and MW were responsible for the statistical analyses and wrote the first draft of the manuscript. MK, MW, JS, and JK performed biochemical analysis. MW, MK, JS, JK, MM, and ZS contributed to the discussion and reviewed and edited the manuscript. All authors contributed to the article and approved the submitted version.

## Conflict of Interest

The authors declare that the research was conducted in the absence of any commercial or financial relationships that could be construed as a potential conflict of interest.

## Publisher’s Note

All claims expressed in this article are solely those of the authors and do not necessarily represent those of their affiliated organizations, or those of the publisher, the editors and the reviewers. Any product that may be evaluated in this article, or claim that may be made by its manufacturer, is not guaranteed or endorsed by the publisher.
